# The wild-type flagellar filament of the Firmicute *Kurthia* at 2.8 Å resolution *in vivo*

**DOI:** 10.1038/s41598-019-51440-1

**Published:** 2019-10-18

**Authors:** Thorsten B. Blum, Sevasti Filippidou, Mathilda Fatton, Pilar Junier, Jan Pieter Abrahams

**Affiliations:** 1 Biology and Chemistry, Laboratory of Nanoscale Biology, Paul Scherrer Institute (PSI), CH-5232 Villigen, Switzerland; 2 Center for Cellular Imaging and NanoAnalytics (C-CINA), Biozentrum, University of Basel, CH-4058 Basel, Switzerland; 3 Laboratory of Microbiology, Institute of Biology, University of Neuchâtel, CH-2000 Neuchâtel, Switzerland; 4 Institute of Biology, Leiden University, Sylviusweg 72, 2333 CC Leiden, The Netherlands

**Keywords:** Proteins, Cryoelectron microscopy

## Abstract

Bacteria swim and swarm by rotating the micrometers long, helical filaments of their flagella. They change direction by reversing their flagellar rotation, which switches the handedness of the filament’s supercoil. So far, all studied functional filaments are composed of a mixture of L- and R-state flagellin monomers. Here we show in a study of the wild type Firmicute *Kurthia* sp., that curved, functional filaments can adopt a conformation *in vivo* that is closely related to a uniform, all-L-state. This sheds additional light on transitions of the flagellar supercoil and uniquely reveals the atomic structure of a wild-type flagellar filament *in vivo*, including six residues showing clearly densities of O-linked glycosylation.

## Introduction

In addition to its crucial role in motility^1^, bacterial flagella also play a key role in adhesion, biofilm formation, host recognition, and invasion^2^. More than 20 genes encode the structural elements of the flagellum^3^. These include a basal body (encompassing the MS, P, and L rings), a motor, a switch (structure composed of FliG, FliM, and FliN, which in *Salmonella* is required to change the direction of rotation of the motor), a hook, a filament, and an export apparatus^4^. The flagellar filament is composed of the protein flagellin that assembles into linear protofilaments. Flagellar filaments of almost all bacteria contain 11 protofilaments, with the exception of *Campylobacter jejuni*, which only has seven^5,6^. Flagellar glycosylation by unusual sugars is essential for motility and may present a novel drug target^7^.

The 11 flagellar protofilaments twist into a supercoil that can vary in rise and handedness, a phenomenon termed polymorphism^8–10^. Each protofilament can exist in the so-called L- or R-state, depending on the conformation of its composing flagellin monomers. In *Bacillus* *subtilis*, the most significant difference between the R-flagellin and the L-flagellin monomers, is a 7.6° tilt of its C-terminal alpha helix, resulting in a reduction in length of R-state protofilaments of about 1.5%, compared to L-state protofilaments^11^. The shorter protofilaments are assumed to run along the inside of the flagellar supercoil. Based on this model, it has been predicted that up to 10 different supercoiled conformations may exist, with the two extreme all-L or all-R states resulting in straight, non-functional filaments^12^. Indeed, when protofilaments of stable, non-interconverting L- and R-conformation mutants are copolymerized at different molar ratios, they form discrete types of filaments from straight (either L- or R-state) to helical (mix of L- and R-states)^13^. When the rotational direction of wild-type flagella reverses – which is required for rapid, random redirection of the bacterium’s path of motion – the handedness of its supercoil flips. Presumably, hydrodynamic drag forces the filament into a new supercoiled ground state. This change in supercoil handedness requires simultaneous flipping of the filament’s helical twist of its protofilaments, implying slight relative shifts of protofilaments and potential redistribution of their L- and R-flagellin conformations.

Thus far, all investigated wild-type, functional flagella contained both L- and R-flagellin protofilaments. The presence of both L- and R-flagellin protofilaments breaks the local, short-range helical symmetry of the flagellar filament, preventing structure determination at high resolution. Only the straight, stiff filaments of non-motile flagellin mutants locked in either the L- or R-conformation have the required ideal helical symmetry for high resolution structural analysis. First the structure of the locked R-type was solved using X-ray crystallography^14^ and cryo-electron microscopy^15^. Afterwards the structure of the locked L-type was solved using cryo-electron microscopy^16^. The best cryo-EM structure so far, is a locked R-type with a resolution of 3.8 Å^17^.

A protofilament can only be running consistently along the inner track of a flagellar super-helix, if (i) it is shorter than its neighbors and (ii) the filament is twisted about the filament’s axis with a helical periodicity identical to that of the super-helix, but with opposite handedness. Axial twists of −1.5° and +4.0° were indeed observed in either all-L or all-R locked, non-motile mutants with straight filaments and uniform protofilament sliding^18^. So far, the structure of wild-type, curved flagellar filaments has not been observed at high resolution. However, when we examined curved, fully functional, wild-type flagella from *Kurthia* sp. strain 11kri321 (here forth called *Kurthia*), we found its flagellin monomers allowed inferring their structure to 2.8 Å resolution. We assumed therefore that they may have existed in one single state.

*Kurthia* is a Gram-positive bacterium belonging to the Firmicute phylum. Both *Kurthia* and *B. subtilis* lack the components of the flagellar P and L rings found in *Escherichia coli*. The annotated genome of *Kurthia* contains all but one of the genes required for the production of a canonical Gram-positive flagellar apparatus. The only missing component is the gene for the dual-function protein FliT. This protein acts as a transcriptional regulator (anti-FlhDC factor) and as a chaperone in the export of the flagellar cap protein FliD^19^. FliT was classified as a fast-evolving component of the flagellar machinery^3^ and could have been missed in the automatic annotation of the genome.

**Figure 1:**
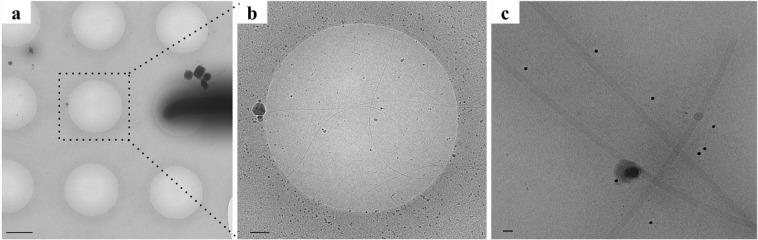
(**a**) Low magnification cryo-EM image of *Kurthia* sp. 11kri321 (dark shade at the middle right-hand side) on a quantifoil grid. (**b**) Zoom-in of a neighbouring hole with flagellar filaments in thin ice and (**c**) flagellar filaments imaged at high magnification. Scale bars: 1 µm, 200 nm and 20 nm.

We analyzed wild-type, mostly curved flagellar filaments projecting from motile *Kurthia* bacteria by transmission cryo-electron microscopy (Fig. 1 and Video S1). We unbiasedly picked filaments with a curvature up to 1.5 rad/µm (Fig. S1). Curvatures were in good agreement with the values measured in wild type *B*. *subtilis* and *E*. *coli*, which have typical average values of 1.25 rad/µm and 1.1 rad/µm, respectively^17,20^. The observed maximum curvature of 1.5 rad/µm was anticipated to induce an additional rotational shift between two flagellin subunits in a protofilament (distance of 5.3 nm) of only 6.1 mrad. This corresponds to a maximal deviation of 0.03 nm, which is 1/10^th^ of the resolution of the reported structure. We therefore considered that it is justified to use helical reconstruction with local refinement of helical parameters and a 2D helical net.

A low-resolution map obtained by sub-tomogram averaging served as an initial reference for helical reconstruction. During subsequent processing, less than 1% of selected helical fragments were discarded. Initially, five 3D classes were identified, which upon helical refinement (including the refinement of the helical parameters) produced good quality potential maps that were so similar that combining the five classes significantly increased the resolution. This suggested that flagellin was essentially present in a single conformation, in which the 11 protofilaments were tilted slightly left by ~ 0.06° (Fig. S2). This 0.06° left-handed helical twist of the protofilaments about the filamental axis is so minute, that the protofilaments could be considered being parallel.

**Figure 2:**
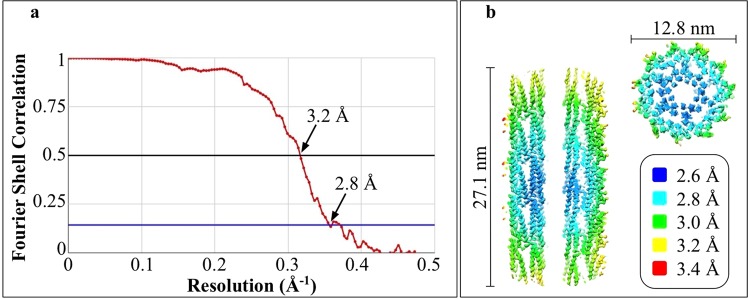
(**a**) Gold-standard Fourier shell correlation (FSC) curves calculated from two independently-refined half-maps, indicate an overall resolution of 2.8 Å at FSC = 0.143. (**b**) Local resolution estimation of the longitudinal section and cross-section of the cryo-EM density map reveals an average resolution better than 2.6 Å in the center of the reconstruction.

**Figure 3:**
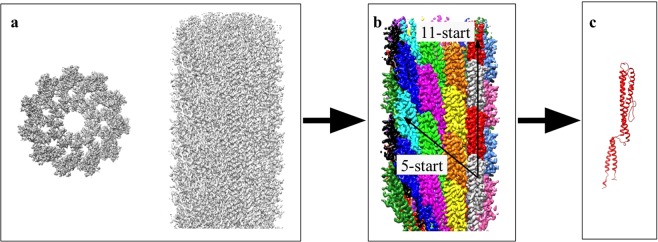
(**a**) Central slice and side view of the EM density. (**b**) Each flagellin was segmented to visualize the 5-start and 11-start helix. (**c**) The EM density of one flagellin was extracted, which was used to build and refine the model.

Helical reconstruction resulted in a map with a maximum resolution of 2.6 Å or better of the most ordered parts and an overall resolution of 2.8 Å (Fig. 2; EMD-10362). The map (Fig. 3a) was segmented into individual flagellin monomers (Fig. 3b). A well-fitting atomic model resulted after auto-building, interactive improvement of the fit and subsequent structure refinement (Fig. S3; PDB ID 6T17). A comparison of the 3D structure of flagellin from *Kurthia* (276 amino acids) and the published structures of L- and R-conformations of *B*. *subtilis* (304 amino acids) show that all share the same overall fold, including five helices and two β-hairpins, but that the loops before and after the β-hairpins were shorter in *Kurthia* (Fig. S4). The interfacial areas of the subunits were analysed using PISA^21^ and the results are similar to *B*. *subtilis*^17^ with one exception: our model suggests a potential additional weak interaction of the C-terminus with the 1-start subunit (Fig. S5). The interacting surface area is small and the density is not well resolved, so interpretation of this additional interaction requires caution.

**Figure 4:**
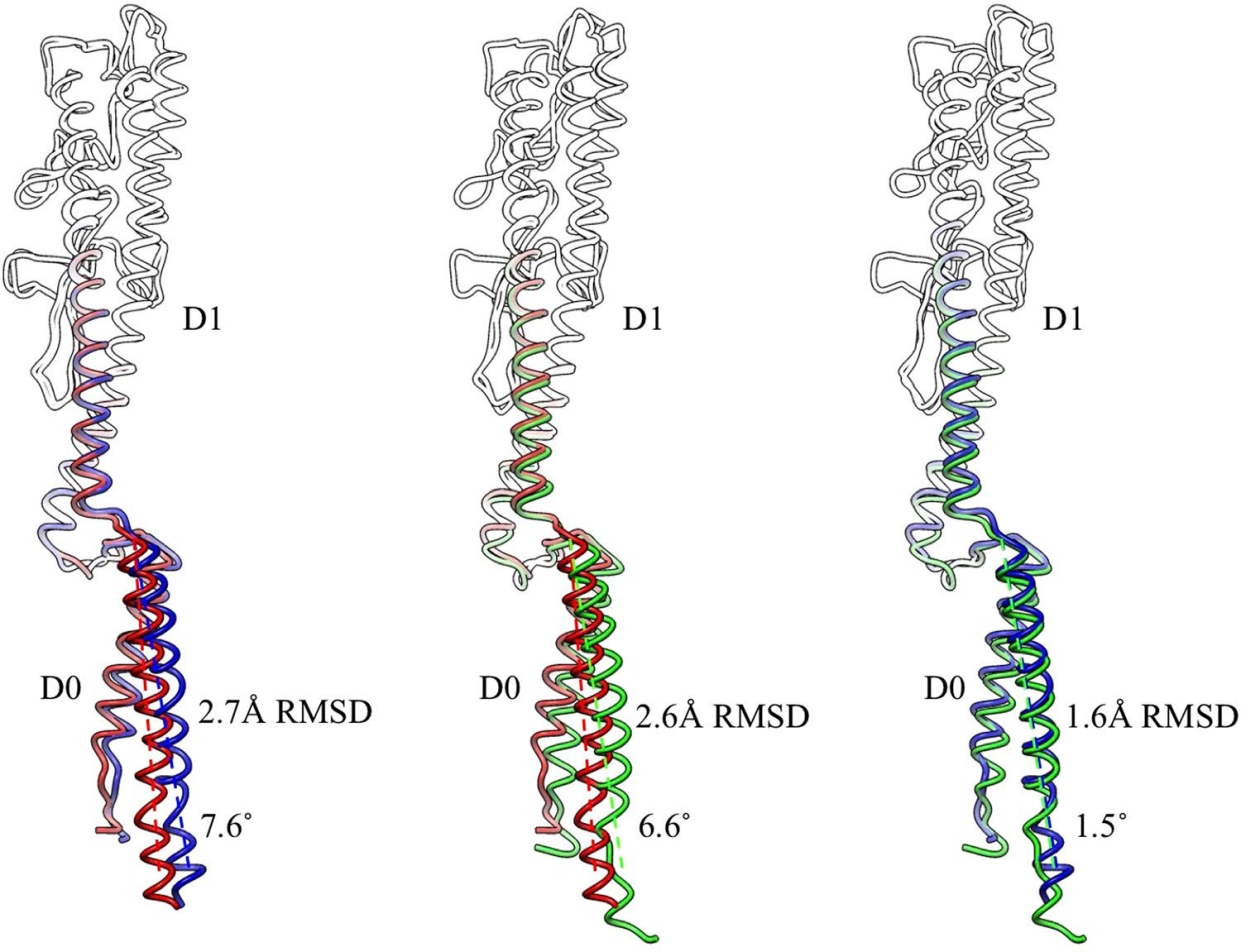
Comparison of the structures of flagellin from *B*. *subtilis* in the L-state (blue) and the R-state (red) with flagellin of *Kurthia* sp. 11kri321 (green), indicates *Kurthia*’s flagellin is in the L-state. The D1 domains were superimposed and the position of the domain D0 was compared. The angle between the helix number 5 in the domain D0 was used to identify the flagellin’s state. The close relationship between the conformations of *Kurthia*’s flagellin and L-state *B*. *subtilis* flagellin is confirmed by the measured RMSD between the D0 domains.

*Kurthia* flagellin was present in a conformation that was most like the L-state as encountered in *B*. *subtilis* (Fig. 4). Despite forcing refinement to include angles between the protofilaments like for *B*. *subtilis* of either L- or R-states, we did not find evidence of their existence in *Kurthia*. However, we cannot fully exclude the possibility that a minority of protofilaments were either in the L- or R-state, since we had to impose helical symmetry to reach a resolution that allowed separating these states. Nevertheless, for the first time, the structure of a bacterially attached, fully functional, wild type flagellar filament could be observed at atomic resolution.

**Figure 5:**
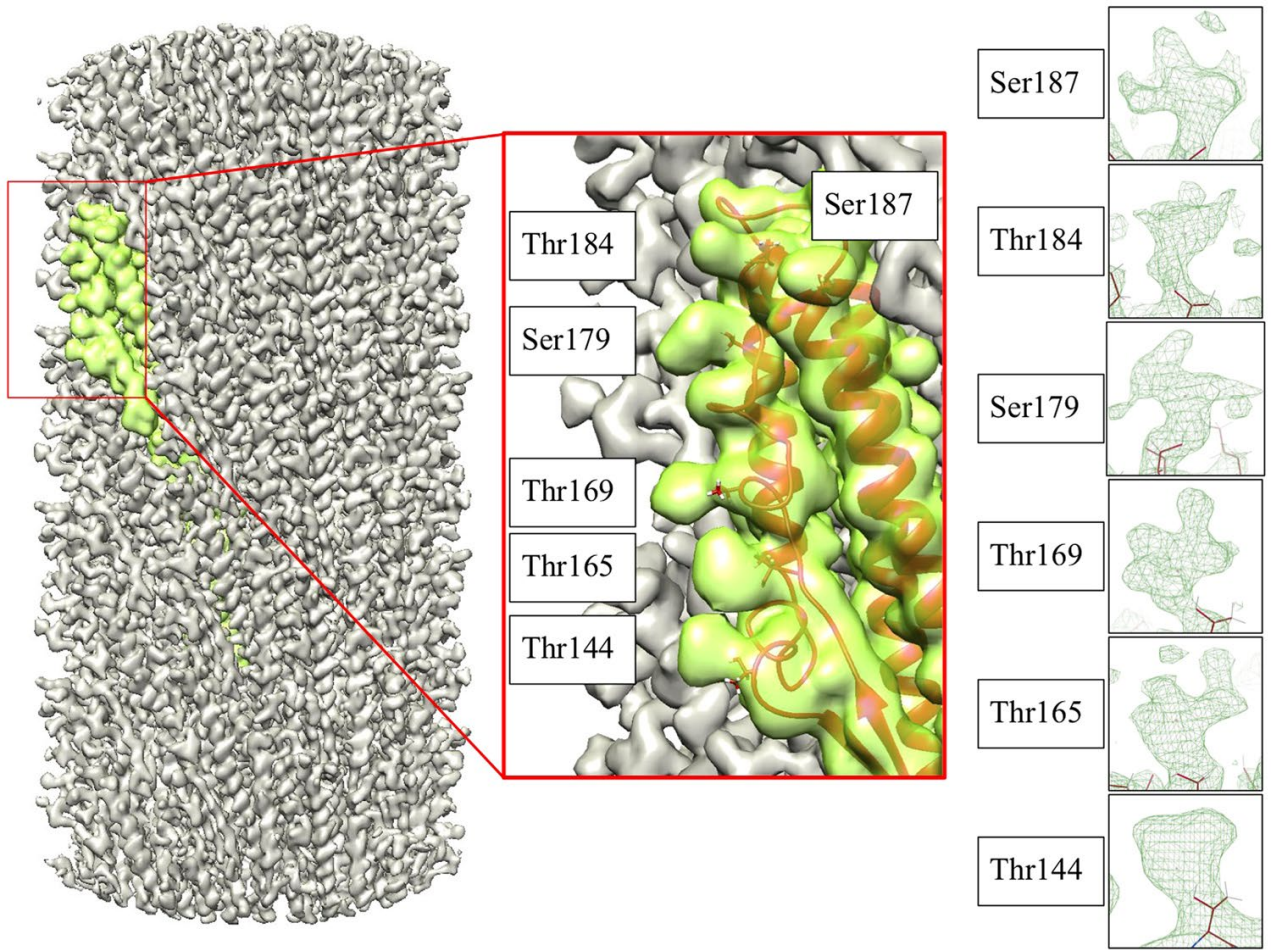
One flagellin monomer was placed into the electron scattering potential map, which showed extra densities corresponding to O-glycosylations of the indicated threonines and serines.

The electron scattering potential map of the functional *Kurthia* filament showed six extra densities corresponding to O-glycosylations of threonines and serines (Fig. 5). These amino acids are not conserved in *B*. *subtilis* (Fig. S4) and glycosylations were absent in published maps of mutated flagellin from *B*. *subtilis*. Although the extra densities clearly correspond to glycosylation, the relatively poor order of these moieties prevented unequivocal identification of their composition and could therefore not be included in the atomic model. Further chemical identification of the sugars would be required for completing the atomic model. However, since *Kurthia* is not considered to be pathogenic, and hence these moieties would not present novel drug targets, we did not further pursue this aspect.

Our data indicate that wild-type *Kurthia* flagellin was present in just one single conformation, and formed nearly parallel protofilaments, irrespective of flagellar curvature. This suggests that in *Kurthia*, the polymorphic switching of the flagellar supercoil from left- to right-handed and *vice versa*, as induced by reversal of the flagellar motor, might not require a conformational change of the flagellin monomers. The persistence length of the *Kurthia* filament was similar to that of active filaments of other bacteria, as the range of curvatures we encountered in the *Kurthia* flagella did not differ significantly from the values reported in other bacteria^17,20^. Nevertheless, we have to consider the possibility that the single conformational state of flagellin in *Kurthia* filaments is an experimental artifact. It may be the case that the thin ice, required for high resolution cryo-EM, forced the filaments from a supercoiled into a closely related flat-curved conformation, in which a singular conformational state is more energetically favorable. In that case, the structure of the flagellar filament that we describe here, might correspond to a transition state between the left- and right-handed flagellar supercoil (or *vice versa*), since the protofilaments run virtually parallel, rather than showing a left- or right-handed twist.

If, however, our results reflect the natural state of *Kurthia* flagellin, some residual flexibility at the lateral and longitudinal interfaces of the single-state flagellin monomers would be essential to accommodate a range of both left- and right-handed flagellar supercoils. The virtually parallel arrangement of *Kurthia*’s protofilaments might in that case be required for equalizing the free energy between the left- and right-handed flagellar supercoils and reducing the transition state free energy during reversal of the supercoil handedness. We do not anticipate this to reduce the persistence length of the filaments in view of the observed curvatures of the *Kurthia* flagella. However, the periodicity and amplitude its helical supercoil would be more variable than in bacteria in which specific helical parameters are favored by polymorphic switching of flagellins and their protofilaments. Increased flagellar flexibility would promote rapid switching of the supercoil handedness upon rotational reversal. Our conjecture therefore hints at a possible explanation of *Kurthia*’s potentially non-standard flagellar structure. If *Kurthia*’s natural environment favors very frequent and rapid tumbling over sustained directional motion, evolutionary pressures might have optimized the kinetics of its flagellar supercoil reversals at the expense of its supercoil rigidity.

## Methods

### Culture conditions

*Kurthia* sp. strain 11kri321 was cultured aerobically on Tryptic Soy Agar (TSA) medium at 37 °C, obtaining distinct beige colonies within 18 hours. This strain also grows on Tryptic Soy Broth (TSB), at 37 °C, with an agitation of 110–130 rpm, overnight.

### Motility test

The swimming motility of *Kurthia* sp. strain 11kri321 was determined according to Rashid *et al*.^22^. Deep soft agar TSA tubes were inoculated with colonies from an overnight TSA culture with a straight needle. The bacteria were able to grow throughout the soft agar tube. Strain 11kri321 grown both on TSA and TSB was also observed under the contrast-phase microscope (Leica DM R, magnification 1000x), in order to verify and confirm the swimming motility assay. A motility movie was recorded by filming the strain 11kri321 in a microfluidic device at Newcastle University (Lucy Eland, ICOS Research, School of Computing Science, Newcastle University, Newcastle upon Tyne NE1 7RU). An overnight nutrient broth (NB) liquid culture was diluted at OD 0.1, and then regrown in NB until reaching OD 0.5–0.6. 2 μl of this bacterial suspension was deposited in a polydimethylsiloxane (PDMS) mold and rapidly covered with a microfluidic agarose chip (low-melting point agarose at 4%, ThermoScientific, Ref. R0801) and a top coverslip. The microfluidic system was sealed by plasma bonding (Harrick Plasma Cleaner, Ithaca, New York, Model PDC-002) and infused overnight with a flux of NB medium^23^. After 12 hours, the NB flux was stopped and we realized a timelapse movie by acquiring several pictures with a microscope (1 frame/second) (Nikon Eclipse, Ti-DH; with Camera QImaging RETIGA 2000R) (Video S1).

### Grid preparation and data collection

1 ml cells were centrifuged for 7 minutes at 5000 g, 10 °C and resuspended in 200 µl TSB medium. Then, 3.5 µl of the cell suspension was mixed with 1 µl Protein A- Gold 5 nm (www.cellbiology-utrecht.nl) and 3.5 µl of this mixture was pipetted onto a glow-discharged UltrAuFoil grid (R 1.2/1.3, Au 300). Grids were blotted for 3 seconds and plunge-frozen in liquid ethane using a Leica EM GP with the environmental chamber set at 80% humidity and 20 °C. Data were acquired on a Titan Krios electron microscope at 300 keV (Thermo Fisher), with a GIF Quantum LS Imaging filter (20 eV slit width) and a K2 Summit electron counting direct detection camera (Gatan).

Tilted data sets and images for helical reconstruction were imaged at a nominal magnification of 105k and 130k respectively, resulting in a calibrated super-resolution pixel size of 0.668 Å and 0.528 Å (physical pixel size of 1.336 Å and 1.058 Å). The defocus was fixed to –1.8 µm for tilted data sets and varied between –0.8 and –1.6 µm for helical reconstruction. Tilted data sets were recorded according to the Hagen scheme with intervals of 3° between −60° and +60° using SerialEM^24^. At each angle a movie (0.9 sec exposure in total, 0.3 sec per frame, 3 frames in total) with a dose rate of ~4.9 e^−^/Å^2^ per second (~1.6 e^−^/Å^2^ per frame) was recorded, resulting in a total exposure time of 37 sec and a total dose of 180 e^−^/Å^2^ on sample.

For helical reconstruction 153 movies were recorded with a total dose of 86 e^−^/Å^2^ per movie (20 sec exposure in total, 0.4 sec per frame, 50 frames in total). The dose rate was ~4.3 e^−^/Å^2^ per second (~1.7 e^−^/Å^2^ per frame).

The Focus software^25^ was used to down-sample the super-resolution micrographs by a factor of two, drift-correct and dose-weight using MotionCor2^26^.

### Cryo-ET and subtomogram averaging

Tomograms were CTF corrected and reconstructed with IMOD^27^. The resulting 3D volumes were binned fourfold to a pixel size of 5.344 Å. Contrast was enhanced with a non-linear anisotropic diffusion filter (NAD^28^). Each flagellum was picked with a few points in 3dmod and the space in between was filled every pixel using addModPts. Using an in-house written script, the particles were aligned to the y-axis and to avoid averaging the missing wedge, each particle was randomly orientated about the y-axis. The predefined Euler angles were written into an initial motive list for PEET and the initial reference model was calculated without alignment search. Particle alignment was refined by a maximum angular search of 20° around Phi and 6° around Theta and Psi and a maximum translational search range of 5 pixels in x, y and z. In 5 iteration steps the refinement parameters were minimized to a maximum angular search of 1.5° around Phi, Theta and Psi and a maximum translational search of 1 pixel. The cutoff frequency of 0.25 with a Gaussian falloff standard deviation of 0.05 was used for the final iteration.

### Helical reconstruction

Helical reconstruction (including local refinement of helical parameters to compensate for deviations due to slight bending of the flagellar filaments) was done with RELION 2.1 and RELION 3.0^29^. We manually picked 957 mainly bent filaments with a curvature between 0.8 and 1.5 rad/µm (Fig. S1) from 138 micrographs, using the helix picker in RELION 2.1. In total, 9300 segments were extracted using a box size of 256 pixels (~271 Å) and an inter-box distance of 50.227 Å (Table 1). Several rounds of 2D classification were executed and bad classes were removed resulting in 9270 segments. The EM map obtained by subtomogram averaging was low-pass filtered to 25 Å and used as initial model for 3D classification. The class with the highest population (1 out of 5 classes corresponding to 65% of the segments) was further used for 3D auto-refine, which resulted in an overall resolution of 3.8 Å. However, because all five obtained 3D classes were in the L-state with similar helical rise and twist, all segments were combined in one class and the overall resolution improved to 3.7 Å (FSC 0.143 criterion). During the post-processing step, a soft-edge mask and an estimated map sharpening B-factor of −88.6 Å^2^ gave a map with a resolution of 3.4 Å (by the FSC 0.143 criterion). In Relion 3.0 the beam tilt values for the entire data set and the defocus for each segment was estimated, another run of 3D auto-refine and Post Process, using a soft-edge mask and an estimated map sharpening B-factor of −42.33 Å^2^, was performed resulting in a map with a resolution of 3.2 Å and 2.8 Å (by the FSC 0.5 and 0.143 criterion; Fig. 2a; EMD-10362). Because the filaments are curved and not straight the resolution gets worse the further you are from the center (Fig. 2b).

**Table 1:** Cryo-EM structure determination and model statistics.

**Data collection**	
Magnification	130 kx
Pixel size (Å)	1.058
Defocus Range (µm)	−0.85 to −1.65
Voltage (kV)	300
Exposure time (s per frame)	0.4
Number of frames	50
Total dose (e/Å)	86
**Reconstitution**	
Box size (pixels)	256
Inter-box distance (Å)	50.227
Asymmetrical units	11
Micrographs	153
Manually picked filaments	957
Initial extracted segments	9300
Segments after 2D classification	9270
Resolution after 3D auto-refine (Å)	3.1
Final overall resolution (Å)	2.8
Estimated map sharpening B-factor (Å^2^)	−42.33
Helical rise (Å)	4.83
Helical twist (°)	65.45
**Atomic model**	
GenBank Accession number	WP_068455577
R.m.s. deviations	
Bond lengths (Å)	0.006
Bond angles (°)	1.148
Validation	
Map CC (local)	0.734
MolProbity clashscore	2.31
Overall score	1.05
Rotamer outliers (%)	0.00
Ramachandran plot	
Favored (%)	97.81
Allowed (%)	2.19
Outliers (%)	0.00

### Model building and refinement

The post-processed map was used for model building and refinement. The starting model was generated by I-TASSER^30–32^ using the *Kurthia* sp. 11kri321 flagellin sequence (WP_068455577). The starting model was placed as a rigid body into the density of one flagellin that was segmented in Chimera^33^ and the predicted model was modified in COOT^34^ to fit precisely in our EM map. The obtained model was refined with Phenix real-space refinement^35^. Both steps were repeated until convergence (PDB ID 6T17). The electron density reflects the amino acid sequence very well and virtually all side chains are visible (Fig. S3). Indeed, Phenix reports a correlation coefficient between the model and the map of 0.734. This is an independent validation of the quality of the map and hence of the imposed helical symmetry.

## Supplementary information

Supplementary Figures

Supplementary Video 1
