# Supplementary material for: The wild-type flagellar filament of the Firmicute Kurthia at 2.8 Å resolution in vivo
Source: Sci Rep. 2019 Oct 18;9:14948. doi: 10.1038/s41598-019-51440-1 (PMC6802091; doi:10.1038/s41598-019-51440-1)
Supplement: Supplementary file 1 — Supplementary Figures [file 41598_2019_51440_MOESM1_ESM.pdf]

The wild-type flagellar filament of the Firmicute *Kurthia* at 2.8 Å resolution *in vivo*

Thorsten B Blum[1,2,*], Sevasti Filippidou[3], Mathilda Fatton[3], Pilar Junier[3], Jan Pieter Abrahams[1,2,4]

Affiliations:

[1]Biology and Chemistry, Laboratory of Nanoscale Biology, Paul Scherrer Institute (PSI), CH-5232 Villigen, Switzerland.

[2]Center for Cellular Imaging and NanoAnalytics (C-CINA), Biozentrum, University of Basel, CH-4058 Basel, Switzerland.

[3]Laboratory of Microbiology, Institute of Biology, University of Neuchâtel, CH-2000 Neuchâtel, Switzerland.

[4]Institute of Biology, Leiden University, Sylviusweg 72, 2333 CC Leiden, The Netherlands.

*Correspondence to Thorsten B Blum, thorsten.blum@psi.ch

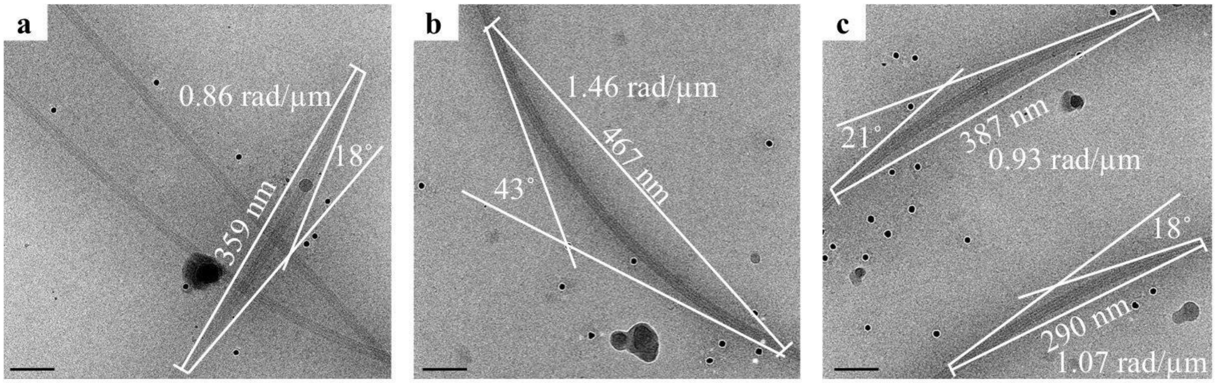

Supplementary Figure 1. The majority of the picked flagella are bent and had a curvature between 0.8 and 1.5 rad/μm. Scale bars: 50 nm.

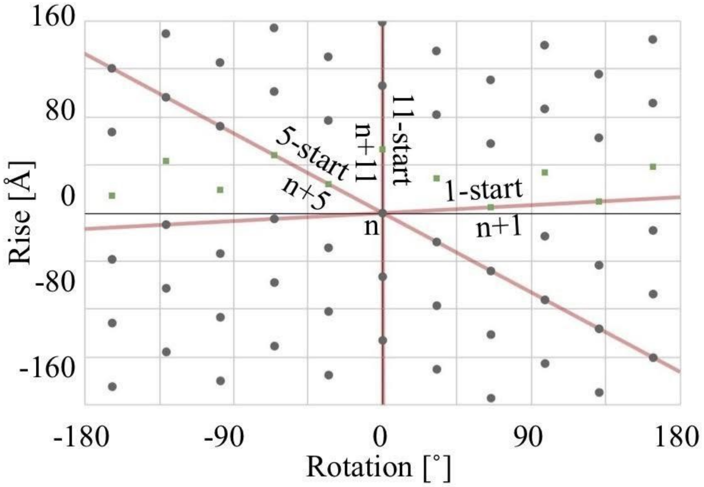

Supplementary Figure 2. Helical net of the flagellar filament. Each point represents one flagellin. One asymmetrical unit containing 11 flagellins is indicated in green. The 11-start helix is tilted by ~ 0.06˚ to the left.

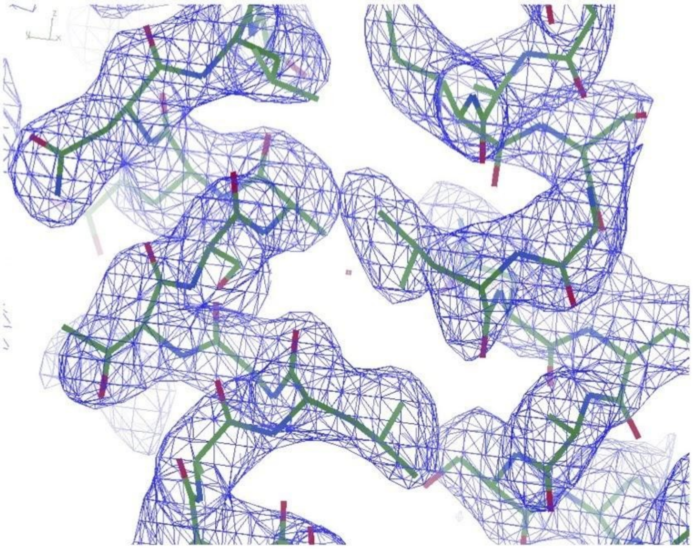

Supplementary Figure 3. Cryo-EM density map with part of the refined model.

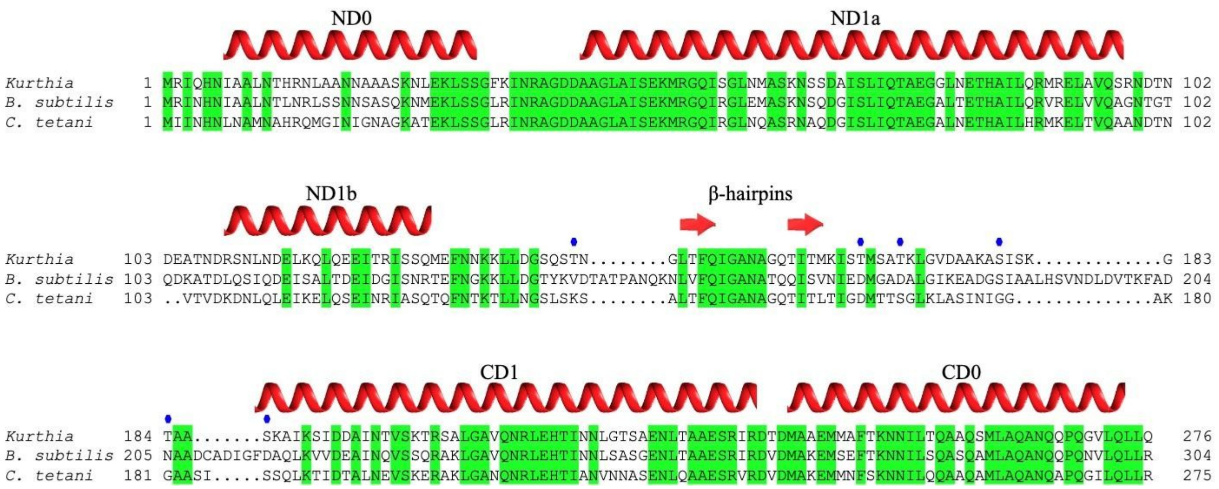

Supplementary Figure 4. Amino acid sequence alignment of the gram-positive bacteria *Kurthia* sp. 11kri321, *Bacillus subtilis* and *Clostridium tetani*. The six O-glycosylations sites of Kurthia are marked by blue hexagons.

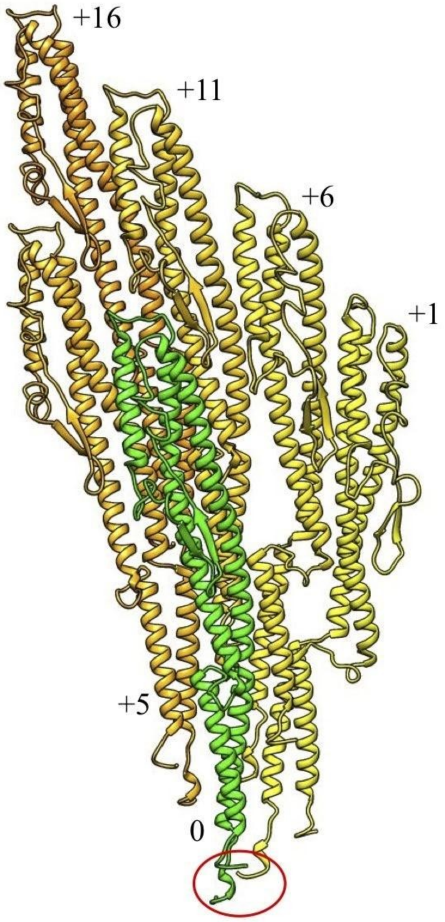

Interfacial subunit-subunit areas

11-start	1947Å^2
5-start	1809Å^2
6-start	569Å^2
16-start	293Å^2
1-start	9Å^2

Supplementary Figure 5. One flagellin subunit (green) with it 5 interacting neighbours (yellow) in the + direction shows a new interaction with the 1-start subunit (red circle). This new interaction is tentative because the C-terminus is flexible and therefore the electron density map is not well resolved.

Supplementary Video 1. Time lapse movie showing the active movement of *Kurthia* sp. 11kri321 cells in a growth chamber in liquid medium.
